# CRISPR/Cas9-Mediated *in vivo* Genetic Correction in a Mouse Model of Hemophilia A

**DOI:** 10.3389/fcell.2021.672564

**Published:** 2021-08-16

**Authors:** Sanchuan Luo, Zhongxiang Li, Xin Dai, Rui Zhang, Zhibing Liang, Wenzhou Li, Ming Zeng, Jinfeng Su, Jun Wang, Xia Liang, Yong Wu, Desheng Liang

**Affiliations:** ^1^Medical Research Institute, Shenzhen Baoan Women’s and Children’s Hospital, Jinan University, Shenzhen, China; ^2^Prenatal Diagnosis Unit, Shenzhen Baoan Women’s and Children’s Hospital, Jinan University, Shenzhen, China; ^3^Hunan Key Laboratory of Medical Genetic, Center for Medical Genetics, School of Life Sciences, Central South University, Changsha, China

**Keywords:** CIRSPR/SaCas9, hemophilia A, intron 1 inversion, gene therapy, gene editing

## Abstract

Hemophilia A (HA), a common bleeding disorder caused by a deficiency of coagulation factor VIII (FVIII), has long been considered an attractive target for gene therapy studies. However, full-length *F8* cDNA cannot be packaged efficiently by adeno-associated virus (AAV) vectors. As the second most prevalent mutation causing severe HA, *F8* intron 1 inversion (Inv1) is caused by an intrachromosomal recombination, leaving the majority of *F8* (exons 2–26) untranscribed. In theory, the truncated gene could be rescued by integrating a promoter and the coding sequence of exon 1. To test this strategy *in vivo*, we generated an HA mouse model by deleting the promoter region and exon 1 of *F8*. Donor DNA and CRISPR/SaCas9 were packaged into AAV vectors and injected into HA mice intravenously. After treatment, *F8* expression was restored and activated partial thromboplastin time (aPTT) was shortened. We also compared two liver-specific promoters and two types of integrating donor vectors. When an active promoter was used, all of the treated mice survived the tail-clip challenge. This is the first report of an *in vivo* gene repair strategy with the potential to treat a recurrent mutation in HA patients.

## Introduction

Hemophilia A (HA) is the most common X-linked inherited hemorrhagic disorder caused by mutations in the *F8* gene, resulting in decreased coagulation factor VIII (FVIII) activity (Batty and Lillicrap, 2019; Ohmori, 2020). Without conventional treatment, hemophiliacs suffer from chronic synovitis caused by recurrent bleeding into joints. In severe hemophilia (FVIII < 1%), hemorrhage into the central nervous system can develop, causing early mortality. Current treatments are based on intravenous infusion of plasma-derived or recombinant FVIII concentrates. As the half-life of circulating FVIII ranges between 14 and 19 h, regular prophylactic intravenous substitution (every 2–3 days) is needed, imposing an economic burden on patients and their families (Batty and Lillicrap, 2019).

Given the monogenic nature of the disorder, in conjunction with the fact that even a slight increase in circulating FVIII (>1%) can markedly improve the quality of life in severe cases, HA has long been identified as an attractive target for gene therapy studies (Liu et al., 2018). Breakthroughs were made by transducing codon-optimized BDD-F8 into hepatocytes using AAV5 vectors (Rangarajan et al., 2017; Pasi et al., 2020). Twenty weeks after treatment, physiological levels of FVIII were achieved in six out of seven participants who received high-dose virus (6 × 10^13^ vg/kg). At the end of a 3-year follow-up study post-treatment, the median FVIII activity remained at 20 IU/dL in the high-dose group. However, it is difficult to obtain life-long therapeutic effects, as the transduced *F8* did not integrate into the genome. Nevertheless, the packaging capacity of AAV was tested to the utmost, given that even the truncated BDD-F8 was 4.4 kb in length. Indeed, because of *F8*’s sheer size, substantially more pre-clinical and clinical studies have been conducted for hemophilia B (HB, an analog of HA). Although HB has a lower occurrence, its therapy gene (*F9*) can be easily packaged by AAV vectors (Nathwani et al., 2011, 2014).

With the rapid development of genome-editing techniques, targeted repair of the mutant gene offers an alternative over gene replacement strategies. There are many advantages for targeted gene repair: (1) in theory, the therapy is lifelong as the endogenous gene is repaired, rather than replaced by an episomal gene cassette. (2) In some cases, only minor modifications are needed to repair a large mutation, making it especially attractive for HA, as the challenge remains to package a functional *F8* using AAV vectors. (3) As gene-editing technology is evolving quickly, targeted gene repair holds great potential for clinical applications. Compared to sporadic mutations, hot-spot mutations are especially worthy of developing targeted gene-repair strategies.

Intron 1 inversion (Inv1) of *F8* is the second most prevalent mutation in patients with severe HA. This intrachromosomal recombination is induced by a 1,041-bp sequence within intron 1 and its reverse repeat located 140 kb telomeric to the *F8* gene. The large inversion completely disrupts *F8* by splitting it into two parts, making it impossible to produce any FVIII protein. Interestingly, the exons 2–26 contain 98% of *F8* coding information (Supplementary Figure 1). In theory, this region could easily be rescued by adding the 146-bp coding sequence of exon 1 and a promoter.

Herein, we established an HA mouse model and tested this therapeutic strategy *in vivo*. After being transduced into hepatocytes using AAV vectors, the coding sequence of exon 1 and a promoter were integrated at the intron 1 locus by using CRISPR/SaCas9, in which two liver-specific promoters and different donor vectors were tested.

## Materials and Methods

### Generation of Hemophilia A Mouse Model

All animal experiments were approved by the Institutional Animal Care and Use Committee at the Jinan University, and animal care was in accordance with the committee’s guidelines. This mouse model was generated in Shanghai Model Organisms Center, Inc. In brief, embryos were collected from the oviducts of superovulated female C57BL/6J mice, after mating to male C57BL/6J mice. Cas9 mRNA and sgRNAs were co-injected into the pronuclei of one-cell embryos. After being cultured in KSOM Mouse Embryo Media overnight, the injected embryos were transplanted into pseudopregnant mice. Genomic DNA was extracted from the tail of new born F0 mice for PCR and sequencing. F1 mice were generated via mating chimeras F0 to C57BL/6J mice.

### Tail Vein Injection of AAV Vectors

The established HA mice are maintained as homozygous female and hemizygous male. All the AAV serotype 8 vectors were produced in Shanghai HANBIO company. Tail-vein administration of AAV vector particles was performed for 8–10-week-old male. AAV8-donor and AAV8-CRISPR/SaCas9 (2 × 10^11^ GC + 4 × 10^11^ GC) were mixed and diluted to 300μL with phosphate-buffered saline plus 0.001% Pluronic F68 before being injected via temporal vein. A low dose of injection (4 × 10^10^ GC + 8 × 10^10^ GC) was also tested.

### Quantitative PCR (qPCR)

Liver tissues were lysised in grinding matrix tubes (MP) using a sample preparation system (MP). Total RNA was isolated using the RNAprep pure Tissue Kit (TIANGEN). Reverse transcription was performed using 1 μg total RNA as template for oligo(dT)_15_-primed RT reaction with Moloney murine leukemia virus reverse transcriptase (Promega, Madison, WI). Equivalent dilutions of the complementary DNA were assessed by PCR for *F8* (primers are 5′-TCAGAGTGATCTGCTCAGTGT-3′ and 5′-TGGGCTT GGCAATGTTGAAA-3′) transcription. A housekeeping gene, *Gapdh* expression was examined to yield a standard representing the mRNA level within the samples (5′-AATGAGAGAGGCCCAGCTAC-3′ and 5′-TGGAAGATGGTG ATGGGCTT-3′).

### CRISPR Design and Efficiency Evaluation

All the sgRNAs were designed using a chopchop online software^1^ (Labun et al., 2016). The sgRNAs were synthesized and cloned into expression vectors (addgene #42230 for SpCas9 and addgene #61591 for SaCas9). We designed 6 sgRNAs for SaCas9 targeting intron1 of *F8* and evaluated their activities in B16-F10 cells before *in vivo* experiments. Briefly, B16-F10 cells were transfected with CRISPR/SaCas9 expression vectors using Lipofectamine 2,000. Genomic DNA was extracted 2 days after transfection. The genomic region encompassing the sgRNA target site was PCR amplified and cloned. After transformation, at least 24 colonies for each transfection were picked up for sequencing.

### Next Generation Sequencing (NGS)

Potential off-target sites of sgRNA1 were predicted using an online bioinformatics tool (Stemmer et al., 2015). Five potential off-target sites were identified in the mouse genome with up to 5 mismatches. Small genomic fragments encompassing these sites were PCR amplified and purified. Deep sequencing was conducted by Sangon Biotech (Shanghai) company. At least 30,000 reads were obtained for each sample. Reference-guided sequence assemblies were performed using an in-house developed assembly platform.

### Immunofluorescence

Mouse liver tissues were fixed in 4% paraformaldehyde for 24 h, then embedded in paraffin. Sections were prepared at 4 μm thickness for immunofluorescence staining. After being boiled in Citrate Antigen Retrieval Solution (BBI Life Sciences) for 3 min, the sections were blocked with 3% BSA, 5% goat serum and 0.1% Triton X-100/PBS for 1 h at room temperature. After an incubation in first antibody (Novus) overnight at 4°C and appropriate wash, the sections were treated with secondary antibody for 1 h at room temperature. DNA was visualized using 4, 6-Diamidino-2-phenylindole (DAPI) and cells were mounted under coverslips using Prolong^TM^ Gold antifade reagent (Thermo Fisher Scientific).

### Tail-Clip Challenge

One month after AAV injection, the distal part of the mouse tail was cut off at the diameter of 1.5 mm. Free-flowing blood for the initial 5 min after tail clip was collected into a citrated microtube. After holding firm pressure on the wound for 1 min, the mice were returned to the cages and examined 48 h later for survival following the hemostatic challenge (Chao et al., 2001).

### Activated Partial Thromboplastin Time (aPTT) Test

Mouse blood samples were collected by removing the eyeballs under anesthesia, mixed with 3.8% sodium citrate anticoagulant at a ratio of 9:1. The samples were spun at 3,000 rpm for 10 min at 4°C, then the upper layer was carefully separated from the red blood cells for the aPTT test using the CA-1500 automatic blood coagulation analyzer (SYSMEX).

### Statistical Analysis

Data were analyzed with GraphPad Prism 8 (GraphPad, San Diego, CA, United States) and represented as mean ± standard deviation (SD). Differences were considered to be statistically significant for *P* < 0.05 (^∗^), *P* < 0.01 (^∗∗^), *P* < 0.001 (^∗∗∗^) and *P* < 0.0001 (^****^).

## Results

### Establishment of an HA Mouse Model

**FIGURE 1:**
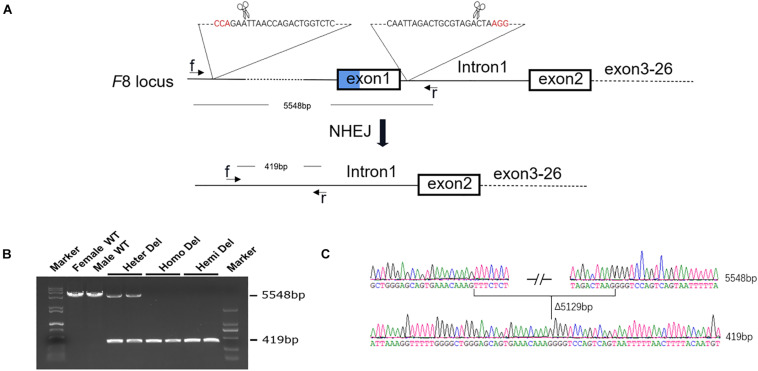
Generation of an HA mouse model. **(A)** Schematic diagram of the strategy for *F8* knock out. Two sgRNAs targeting intron 1 of *F8* and ∼5 kb upstream to the transcription start site, respectively, were designed. A 5,129-bp fragment, including exon 1 and the promoter region, was excised from the genome. **(B)** PCR analysis for detection of the deletion. Using the primers f and r in the schematic, a 5548-bp fragment was amplified from the WT genome. After deletion, the PCR product was 419 bp in length. Both PCR products could be amplified from the female with only one allele deleted. **(C)** Sanger sequencing confirmed the deletion and showed no indels formed in the junction. WT, wild type; Heter Del, heterozygous deletion; Homo Del, homozygous deletion; Hemi Del, hemizygous deletion.

The human Inv1 mutation is caused by recombination during male meiosis between a 1,041-bp sequence within *F8* intron 1 and a reverse repeat outside the gene. There are no similar repeats in intron 1 of murine *F8*, making it difficult to establish a completely identical mouse model for human Inv1 mutation. However, exact modeling of the chromosomal inversion is not necessary for testing our gene repair strategy. In this study, we rescued the exon 2–26 region by adding the missing coding sequence and a promoter. As exon 1 and the promoter part play roles in neither modeling nor the repairing process, we simply excised these parts from the murine genome using CRISPR/Cas9 (Figure 1A).

**FIGURE 2:**
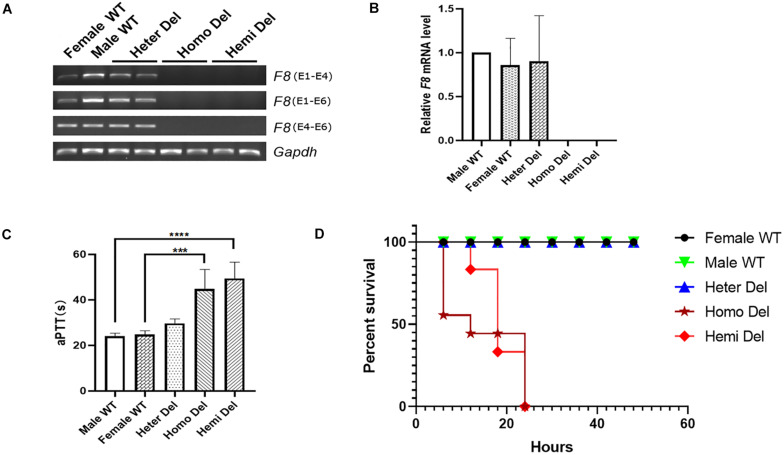
Characterization of the HA mouse model. **(A)** RT-PCR analysis revealed that neither the transcripts cross the exon 1- 2 junction nor the exon 4–6 transcripts could be detected in the knockout mice. *Gapdh* was used as a loading control. **(B)** Expression levels of *F8* mRNA in the hepatic tissue of indicated mice. **(C)** Measurement of coagulation activity by aPTT in mice at 2 months of age. **(D)** Survival rate of mice after tail clip challenge. The mice were monitored for 48 h after tail clipping. Male WT, *n* = 10; Heter Del, *n* = 10; Homo Del, *n* = 8; Hemi Del, *n* = 6. ^∗∗∗^*P* < 0.001, ^****^P < 0.0001.

After injection of CRISPR/Cas9 components into mouse zygotes, a mouse strain was identified by PCR and sequencing (Figure 1B). A 5,129-bp fragment including *F8* exon 1 and the promoter region was deleted from the genome. No insertions or deletions (indels) were detected at the junction (Figure 1C). *F8* mRNA was undetectable in homozygous or hemizygous-deleted mice (Figures 2A,B). The activated partial thromboplastin times (aPTTs) of homozygous or hemizygous-deleted mice were significantly increased (44.88 ± 3.81 s and 49.46 ± 3.19 s, respectively), compared to wild type (WT) mice (24.08 ± 0.57 s). The aPTTs of heterozygous-deleted female carriers were not prolonged (Figure 2C). The mice were also subjected to a tail-clip challenge. All of the homozygous and hemizygous knockout mice died within 24 h, while all of the WT mice and female carriers survived (Figure 2D). These results demonstrated that the mouse strain established in this study is an appropriate model for HA.

### Molecular Correction for the Truncated *F8* Gene *in vivo*

**FIGURE 3:**
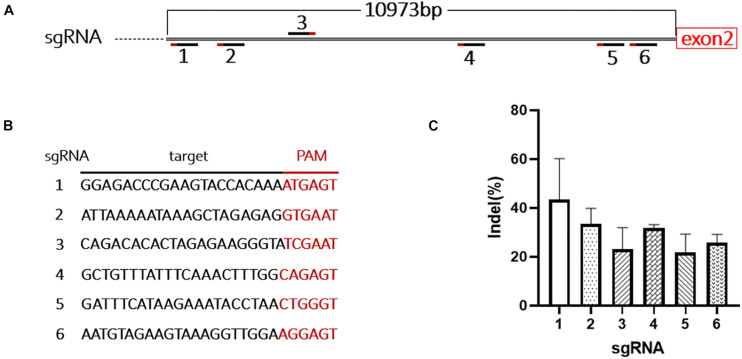
Design and evaluation of the sgRNAs. **(A)** Schematic shows the location of the 6 sgRNAs targeting intron 1 of *F8*. **(B)** Target sequences and PAMs of the 6 sgRNAs. **(C)** The cutting activity of each sgRNA was evaluated in B16-F10 cells, the experiment was performed in triplicate.

Due to the limited packaging capacity of AAV vectors, the Staphylococcus aureus Cas9 (SaCas9) system was used to promote integration efficiency (Ran et al., 2015). During mouse modeling, only 181 bp of intron 1 was excised at the 5’ end, leaving most of intron 1 (10,973 bp) in the genome (Figures 1A, 3A). We designed six sgRNAs targeting this region and tested their efficiency in mouse B16-F10 cells (Figure 3B). The most efficient sgRNA (42 ± 9%) was used in the following experiments (Figure 3C and Supplementary Figure 2).

**FIGURE 4:**
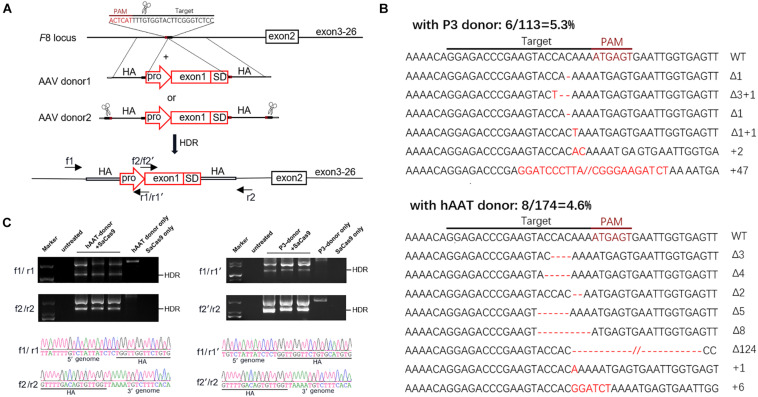
Targeted repair of the truncated *F8 in vivo*. **(A)** Schematic representation of the repair strategy for the truncated *F8*. HA, homologous arm; pro, promoter; SD, splice donor site. **(B)** Target sequences and the mutation induced by CRISPR/SaCas9. The wild-type (WT) target sequence is shown at the top with the intended target site of the sgRNA. Deletions are indicated by red dashes, and insertions by red capital letters. **(C)** PCR analysis for detection of the targeted integration. The expected product for HDR integration is indicated. The PCR products were confirmed by Sanger sequencing. Larger products corresponding to NHEJ integration were also sequenced (see Supplementary Figure 5).

The donor vectors were designed to facilitate targeted integration precisely at the double-strand breaks (DSBs) generated by sgRNA1. A liver-specific promoter, the 146 bp-coding sequence of exon 1 and the splice donor site were packaged within a 1,501-bp homologous sequence (AAV donor type 1). It has been reported that a much higher integration efficiency in hepatocytes can be achieved by *in vivo* excising of backbone sequences from the donor vector, compared to classic homologous recombination (Yao et al., 2017). To test this, we constructed another type of donor, by flanking the type 1 donor with two opposing sgRNA1 sites (AAV donor type 2). We used two liver-specific promoters in this study (Figure 4A): a very small and robust liver-specific promoter designated as P3 designed *in silico* (minimal transthyretin promoter, coupled to a *de novo* designed hepatocyte-specific *cis*-regulatory module 8), and a modified hAAT promoter, used successfully in previous HB gene therapy studies (Supplementary Figures 3, 4; Nathwani et al., 2006; Viecelli et al., 2014).

Six weeks after AAV vector injection, the genomic DNA of the liver tissues was extracted and analyzed. We evaluated the *in vivo* cleavage efficiency by sequencing the T-A clones of the PCR-amplified genome sequence. We found that the indel ratio in mice receiving donors with the P3 promoter (6/113 = 5.3%) and the hAAT promoter (8/174 = 4.6%) were comparable (Figure 4B). Next generation sequencing (NGS) was conducted and the liver DNA from the mice receiving AAV vectors yielded 3.14% indels at the target sites. To evaluate the potential off-target effects caused by CRISPR/SaCas9, we predicted 5 off-target sites in the mouse genome with up to 5 mismatches using an online bioinformatics tool (Stemmer et al., 2015). None of the sites showed obvious cleavage compared to the untreated mice (Table 1 and Supplementary Table 3).

**TABLE 1:** On-target and off-target analyses by NGS.

Site	Target (mismatches in red)	PAM (NNGRRT)	Gene	Site position	Indel%	
					Untreated	Treated
On-target	GGAGACCCGAAGTACCACAAA	ATGAGT	*F8*	Intronic	0.09	3.14
Off-target 1	GGAGACC**TT**AAGT**CA**CACAAA	TGGAAT	*Bc017158*	Intergenic	0.15	0.15
Off-target 2	**A**GA**T**ACC**A**GAAGTACCACA**C**A	CTGAGT	*Gm25798*	Intergenic	0.07	0.09
Off-target 3	GG**T**GACCC**T**AA**A**TACCACAA**G**	GGGAGT	*Abcb4*	Exonic	0.17	0.16
Off-target 4	G**CT**G**T**CC**T**GAA**A**TACCACAAA	ATGAGT	*Gm25693*	Intergenic	0.16	0.13
Off-target 5	**CT**AGA**G**C**A**GAAGTA**A**CACAAA	GAGGGT	*Dsg3*	Intergenic	0.14	0.13

*On-target at the F8 locus and 5 potential off-target sites with up to 5 mismatches were evaluated. Treated means mice received AAV vectors (n = 3).*

We further examined the integration by PCR and Sanger sequencing. Homology-directed repair (HDR) was confirmed in all mice that received both donors and CRISPR/SaCas9. Beyond the HDR band, a larger product was also amplified (Figure 4C). Sanger sequencing of the products confirmed that the donor vectors were also integrated at the target loci in a non-homologous end joining (NHEJ) manner (Supplementary Figure 5B). Meanwhile, integration via NHEJ in the reverse orientation was also detected (Supplementary Figures 5C,D).

**FIGURE 5:**
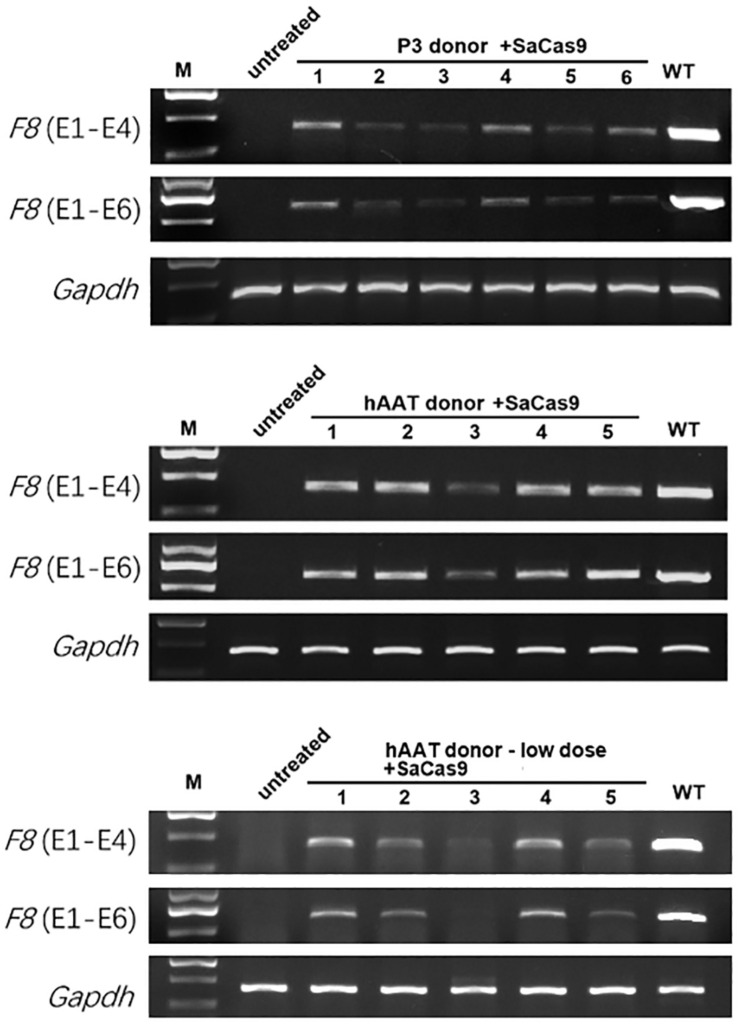
RT-PCR analysis of the liver tissues 1 month after treatment. *Gapdh* was used as a loading control.

**FIGURE 6:**
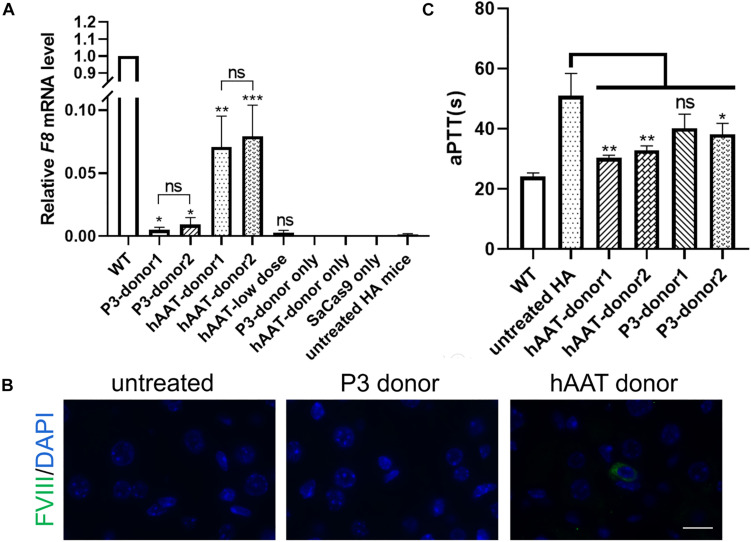
*F8* expression after targeted gene repair. **(A)** Expression levels of *F8* mRNA in the hepatic tissue of indicated mice 1 month after AAV injection. The analysis was performed in triplicate. **(B)** Immunofluorescence staining of FVIII in liver sections of indicated mice. **(C)** Measurement of coagulation activity by testing the aPTT of the plasma collected from the indicated mice 1 month after AAV injection. ^∗^*P* < 0.05, ^∗∗^*P* < 0.01, ^∗∗∗^*P* < 0.001.

RT-PCR of mRNA extracted from liver lysates was performed to examine the transcription after genetic correction. Although *F8* was not transcribed in untreated HA mice, transcripts could be detected in all of the groups that received both CRISPR/SaCas9 and donor vectors, regardless of donor-vector type (Figure 5). The amplified bands were confirmed by Sanger sequencing (Supplementary Figure 5). However, the mRNA level was substantially different among the experimental groups. Quantitative PCR of the cDNA revealed increases in the *F8* mRNA from undetectable to 0.5–0.9% and 7.1–7.9% of WT mRNA levels for mice treated with P3 donors and hAAT donors, respectively. A previous paper reported that removing the backbone from the donor vectors *in vivo* can efficiently enhance the integration rate, especially in hepatocytes transduced by AAV vectors (Yao et al., 2017). However, based on the mRNA levels in this study, the efficiency was not significantly improved when using type 2 donors. When using the two types of donor vectors, the mRNA levels were 0.5 ± 0.1% vs. 0.9 ± 0.2% (*P* = 0.1935) for the P3 promoter and 7.1 ± 1.2% vs. 7.9 ± 1.1% (*P* = 0.6225) for the hAAT promoter (Figure 6A). Immunostaining showed that sporadic cells (< 1%) expressed FVIII in liver tissues after receiving donor vectors with the hAAT promoter. However, likely because the P3 promoter is less active, FVIII protein was undetectable in cells repaired by P3 donors (Figure 6B).

### Restoration of Hemostasis After Treatment

To explore whether the molecular correction can restore the clotting activity, we evaluated the clotting function by measuring the aPTT. The average aPTTs for WT and untreated HA mice were 24.08 ± 0.57 s and 51.02 ± 3.03 s, respectively. One month after injection of CRISPR/SaCas9 and donor vectors with hAAT promoter, the aPTTs dropped significantly to 30.33 ± 0.51 s and 32.88 ± 0.76 s, respectively, for type 1 and type 2 donors. For the groups in which P3 promoter was used, the aPTTs were improved to 40.13 ± 2.75 s and 38.18 ± 1.84 s, respectively, for type 1 and type 2 donors (Figure 6C).

**FIGURE 7:**
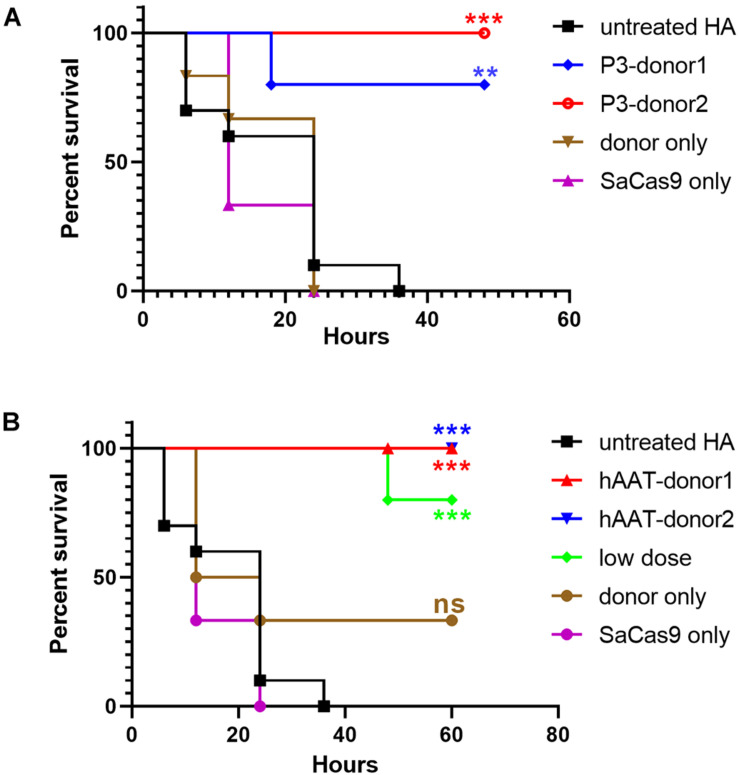
Proportions of surviving mice after the tail-clip challenge. **(A)** Mice treated with donor vector using P3 promoter. Untreated HA, *n* = 10; P3-donor 1, *n* = 5; P3-donor 2, *n* = 5; donor only, *n* = 6; SaCas9 only, *n* = 6. **(B)** Mice treated with donor vectors using hAAT promoter. Untreated HA, *n* = 10; hAAT-donor 1, *n* = 5; hAAT-donor 2, *n* = 5; low dose, *n* = 5; donor only, *n* = 6; SaCas9 only, *n* = 6. Each experimental group was compared with the untreated group using the log-rank test. ^∗∗^*P* < 0.01, ^∗∗∗^*P* < 0.001.

To evaluate whether the bleeding phenotype was improved, the mice were subjected to a tail-clip challenge 1 month after AAV injection. Nearly all of the mice that received either donor vector or CRISPR/SaCas9 only died within a 48-h observation period. Only two of the mice treated with hAAT donor only survived (*n* = 6). However, the survival was significantly improved in all groups that received both CRISPR/SaCas9 and donor vectors. In particular, all of the mice that received hAAT donors and CRISPR/SaCas9 survived during the test, regardless of donor-vector type. The results showed no difference between the two types of donor vectors (Figure 7).

As the groups using hAAT promoter performed better, we tried to reduce the AAV dosage by 80%. One month after injection of low-dose vectors with hAAT promoter, RT-PCR revealed that *F8* transcription was restored (Figure 5, bottom). Although the expression level was very low, survival improved significantly in the tail-clip challenge (Figures 6A, 7B).

## Discussion

Since the first clinical trial 30 years ago, introduction of a functional gene has become the main strategy for gene therapy studies (Anderson et al., 1990). In recent years, the development of programmed nucleases has revolutionized targeted gene-editing technology (Waugh and Sauer, 1993; Miller et al., 2011; Ran et al., 2015). Meanwhile, an increasing number of *in vivo* studies have selected AAV vectors rather than retroviral or adenoviral vectors to transduce therapeutic genes. It is possible to develop safe and efficient gene therapy strategies by combining the above. However, because of their limited capacity, it is inefficient to transduce large fragments by AAV vectors, compromising the efficacy of this combination. It is worthwhile to develop methods to repair the mutated gene rather than replace it with a functional one, at least for some hot-spot mutations. These approaches proved to be more efficient and reasonable in certain cases (Hu et al., 2019; Zhou et al., 2020).

Based on a meta-analysis of the prevalence of hemophilia in males, the expected number of patients with severe HA worldwide is 353,000 (Iorio et al., 2019). The frequency of Inv1 in severe hemophiliacs ranges from 1 to 8%, as reported in different populations (Salviato et al., 2004; Pinto et al., 2016). In most reports, fewer than 5% of severe HA cases are caused by Inv1. If we expected the frequency ranges from 2.5 to 5%, there would be 8,800–17,500 severe hemophiliacs caused by Inv1 worldwide. Moreover, one-third of HA cases occur sporadically, without a familial history (Graw et al., 2005). Inv1 cannot be screened via current methods of prenatal genetic testing including G-banding, CMA, or even WES, as this homologous recombination does not alter any bases in the genomic sequence. In other words, it is difficult to prevent the birth of new patients with this mutation.

Park et al. reported direct reversion of the 140 kb fragment of INV1 in ES cells and patient-derived iPSCs. Four corrected iPSC clones were obtained by transient expression of CRISPR/Cas9. Endothelial cells differentiated from corrected iPSCs expressed the *F8* gene and functionally rescued factor VIII deficiency in a mouse model of hemophilia (Park et al., 2014, 2015). However, it is a challenge to revert such a large fragment *in vivo*.

The liver has long been considered the major site of FVIII production. Recent studies have shown that FVIII is synthesized in endothelial cells, whereas hepatocytes produce no detectable FVIII—likely attribute to its molecular chaperon vWF, which is synthesized exclusively in endothelial cells and megakaryocytes (Everett et al., 2014; Shahani et al., 2014; Wang et al., 2020). Although *F8* is transcribed in many cell types including hepatocytes, only vWF-expressing cells can physiologically secrete FVIII. However, in this study, ectopic expression of *F8* in hepatocytes was sufficient to shorten the aPTT and improve the survival of HA mice. Hepatocyte is an attractive cell type for transgene production and secretion. Several groups reported successful hemophilia A amelioration by targeting the liver-expressed mouse *Alb* locus *in vivo* (Sharma et al., 2015; Chen et al., 2019; Zhang et al., 2019). AAV vectors were used to transfer the transgene and artificial nucleases into the liver. Having been integrated into the chromosome *via* NHEJ, the promoter-less BDD-F8 was expressed under the robust *Alb* promoter. Chen et al. reported that therapeutic level of plasma FVIII was achieved even 7 months after injection. Conversely in this study, we integrated a foreign promoter to the endogenous *F8* coding sequence. In order to repair *F8*, the sizes of integrated fragment (597–1,002 bp) were much smaller than BDD-F8 (more than 4 kb) used in these studies. Furthermore, the donors were integrated into the genome via HDR and NHEJ simultaneously. Together, the results showed that FVIII could be produced by hepatocytes once driven by a robust promoter. It might be interesting to target endothelial cells, especially the liver sinusoidal endothelial cells *in vivo*. To the best of our knowledge, no one has tried to repair *F8* in endothelial cells *in vivo*. The HA mouse model established in this study is very suitable for such research in the future.

We used two liver-specific promoters in this study and designed two types of donor vectors for each promoter. Beyond the classic donor for HDR (type 1), we generated another type of donor (type 2), by flanking the type 1 donor with two additional target sites for the sgRNA. However, this attempt did not significantly improve the gene expression levels (Figure 6A). In contrast, the mRNA level in the hAAT group was approximately 10 times higher than that in the P3 group. Devoid of CRISPR/SaCas9, all of the mice receiving only the P3 donor died (*n* = 6), while two of the mice that only received the hAAT donor survived in the tail clip challenge (*n* = 6) (Figure 7). These results coincide with a previous study that integrated a promoterless *F9* within the mouse albumin gene to ameliorate the bleeding diathesis in HB mice without using artificial nucleases (Barzel et al., 2015). However, the AAV dosage was higher than that used in the current study. In fact, the integration with the hAAT donor was probably less efficient than with the P3 donor, as the integrated fragment of the hAAT donor (1,002 bp) was longer than the P3 donor (597 bp). It is unclear why the efficiency of P3 promoter was approximately 1/10 lower than the hAAT promoter, based on the qRT-PCR results in the present study. P3 promoter was proved to be robust and liver-specific. It has also been successfully used in gene therapy and mouse modeling studies (Nair et al., 2014; Viecelli et al., 2014; Singh et al., 2018). In these studies, vectors were injected as minicircle naked-DNA or AAV particles. The vectors are multi-copy and episomal in liver cells. However, in the present study, promoter was integrated into the X chromosome with only one copy in males. *F8* could not be transcribed *via* residual episomal P3 promoter. These data suggest that the selection of an appropriate promoter is a particularly important factor in our repair strategy. Although we only tested two promoters, a substantial improvement might be achieved when a more active liver-specific promoter was used.

Effective cleavage of the target site is a prerequisite for highly efficient integration. In this study, only ∼3.14% of indels were achieved *in vivo* (Table 1). To the best of our knowledge, when a better sgRNA is used, 50% of cleavage in the mouse liver can be induced by CRISPR/SaCas9 delivered by AAV vectors (Ohmori et al., 2017; Singh et al., 2018). If the current repair approach is applied to human Inv1, the residual part of intron 1 adjacent to exon 1 would be suitable to integrate the repairing DNA fragment. This part is 6506 bp in length that would be sufficient for the design of a more active sgRNA (Supplementary Data 1).

Beyond HDR, the donor was also integrated into the genome via NHEJ in this study, although the absolute efficiencies of HDR and NHEJ could not be quantified by our PCR-based analysis. NHEJ has long been recognized as error-prone, but recent studies have also demonstrated its intrinsic precision (Bétermier et al., 2014; Suzuki et al., 2016). NHEJ is useful when foreign DNA is integrated into intronic region. The contribution of NHEJ for therapy should not be underestimated, as the majority of hepatocytes remain in the quiescent phase. However, NHEJ also conducts useless integrations. For example, donors can be integrated in reverse orientation during NHEJ. Moreover, when the type 2 donor is used, the released AAV backbone is also integrated. It is possible to improve the integration by controlling the orientation and removing the backbone sequence from the donor (Suzuki et al., 2016).

Expression levels and integration efficiency can be improved via the methods described above, but it might be more attractive to decrease the viral dosage. In this study, with less than 1% of the hepatocytes repaired, aPTT was shortened and all of the mice survived the tail-clip challenge. The survival of the experimental group that received much lower quantities of AAV vectors (4 × 10^10^ GC + 8 × 10^10^ GC) was significantly improved too. In our opinion, there is no need to pursue a very high level of FVIII at the beginning of targeted gene repair. Once the endogenous gene is repaired in the genome, it will not attenuate like other strategies that transduce an episomal cassette. Furthermore, it would be safer and more cost-effective if clinical symptoms can be improved by low-dose AAV vector administration. After all, for patients with severe HA, a slight increase in circulating FVIII can markedly improve the quality of life.

## Conclusion

In this study, we generated an HA mouse model by excising the promoter and exon 1 of *F8* from the genome. Thereafter, the truncated *F8* was repaired via intravenous injection of AAV vectors with repairing DNA sequence. After treatment, *F8* expression was restored and aPTT was shortened. When a proper promoter was used, the survival rate was increased even when a much lower dose of AAV vectors was administered. To the best of our knowledge, this is the first report of an *in vivo* gene repair strategy with the potential to treat a recurrent mutation in HA patients.

## Data Availability Statement

The raw data supporting the conclusions of this article will be made available by the authors, without undue reservation.

## Ethics Statement

The animal study was reviewed and approved by Animal Care and Use Committee of Jinan University.

## Author Contributions

YW and DL initiated and designed the project. SL, ZhoL, XD, RZ, ZhiL, WL, MZ, and JS performed the experiments. JW and XL performed the statistical analysis. SL and YW wrote the manuscript. DL and ZhoL reviewed the manuscript. All authors contributed to the article and approved the submitted version.

## Conflict of Interest

The authors declare that the research was conducted in the absence of any commercial or financial relationships that could be construed as a potential conflict of interest.

## Publisher’s Note

All claims expressed in this article are solely those of the authors and do not necessarily represent those of their affiliated organizations, or those of the publisher, the editors and the reviewers. Any product that may be evaluated in this article, or claim that may be made by its manufacturer, is not guaranteed or endorsed by the publisher.

## Footnotes

1
http://chopchop.cbu.uib.no/
