# Supplementary material for: CRISPR/Cas9-Mediated in vivo Genetic Correction in a Mouse Model of Hemophilia A
Source: Front Cell Dev Biol. 2021 Aug 16;9:672564. doi: 10.3389/fcell.2021.672564 (PMC8415270; doi:10.3389/fcell.2021.672564)
Supplement: Supplementary file 1 [file Data_Sheet_1.DOC]

***Supplementary Material***

**Supplementary Tables**

**Table S1. Primers used in this study.**

| Screening of mouse modeling | f | GGAATATCAAAGTCCCACTGAAAAC |
| --- | --- | --- |
|  | r | AGGAGACCCGAAGTACCACAAA |
| Detecting of integration | f1-1 | TGTGAGCTAGATTCCTACCTGAG |
|  | f1-2 | CTGCATTAAAGGGGCATAGC |
|  | f2-1 | TTGCTCCTCCGATAACTGGG |
|  | f2-2 | TTGCTCCTCCGATAACTGGG |
|  | f2′-1 | CGTCTGTCTGCACATTTCGT |
|  | f2′-2 | CAGGTTTGGAGTCAGCTTGG |
|  | r1-1 | CTAAACCACGCCAGGACAAC |
|  | r1-2 | TGCTCCTCTCCACCGAAATT |
|  | r1′-1 | GCTCAGAAAGAAGCAAGCGA |
|  | r1′-2 | GCTTGTGGATCTGTGTGACG |
|  | r2-1 | AGACACAGGAATTTAAGGGCG |
|  | r2-2 | AGAGAGGTGAATGTGGTGGTG |
|  | LHA-r | TGGTCTGTTTTCAGTGGGACT |
|  | RHA-r | ACAGGAGACCCGAAGTACCAC |
| RT-PCR for *F8* | Forward based on exon 1 | TCAGAGTGATCTGCTCAGTGT |
|  | Forward based on exon 4 | TGACCCTCCATGTCTCACTTAC |
|  | Reverse based on exon 4 | GTAAGTGAGACATGGAGGGTCA |
|  | Reverse based on exon 6 | GTGCATTTTAGGCCAGTCTCT |
| RT-PCR for *Gapdh* | Forward | AACGACCCCTTCATTGACCT |
|  | Reverse | CCCTTCCACAATGCCAAAGT |

**Table S2. Primers used in evaluation of sgRNAs.**

| For sgRNA | Forward primer | Reverse primer | Expected product size, bp |
| --- | --- | --- | --- |
| 1 | AAAATCCTCTGTCAAGTGCAAC | TTGGAGTTCTGGGCCATTCT | 704 |
| 2 | TGCGTGTTGGCTATCAGAGT | CACAGGAATTTAAGGGCGTGT | 716 |
| 3 | CCCACATCCTCACCAGAACT | GGAAACGCTCAGGAGGTCTT | 774 |
| 4 | AGCTCCAGGTTCAGTGTCAA | GGCAGTGTTCCAAGGTTCAC | 778 |
| 5 | GGAAGAGCAGGCCAGTAGAA | AATTCTGTCCTTCCCTGCCA | 608 |
| 6 | GTGGAGTGAAATTTGGGGCT | CCATGACAGAGCTACCCAGT | 762 |

**Table S3. Primers used in next generation sequencing.**

| site | Forward primer | Reverse primer | Expected product size, bp |
| --- | --- | --- | --- |
| On-target | TGGGAGCAGTGAAACAAAGG | CAGCAAGATACCACCAGATA | 309 |
| Off-target 1 | AACCTAATGGGTGGTCAATC | TTCCCACATAACCAACTTTT | 255 |
| Off-target 2 | CATGGCTACCTATTTCTTCC | CTCCCTGATGCTCACTTCTA | 287 |
| Off-target 3 | TTAACCTAACTGGGTGAAAG | TGTGCCACCTGAACTGTTTC | 273 |
| Off-target 4 | GTAAATGGCATACTGAAGTG | TATCCTCTGATCTCCTGGGT | 294 |
| Off-target 5 | AAGAAGGAAGACGGGAGAAA | GCACATTGAATAAGGGCTGT | 339 |

**Supplementary Figures**


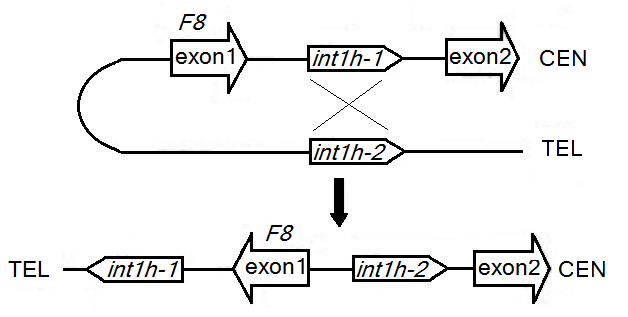


**Figure S1.** Diagram of *F8* intron 1 inversion**.** This inversion is the result of a homologous recombination induced by two repeats (int1h-1 and int1h-2).


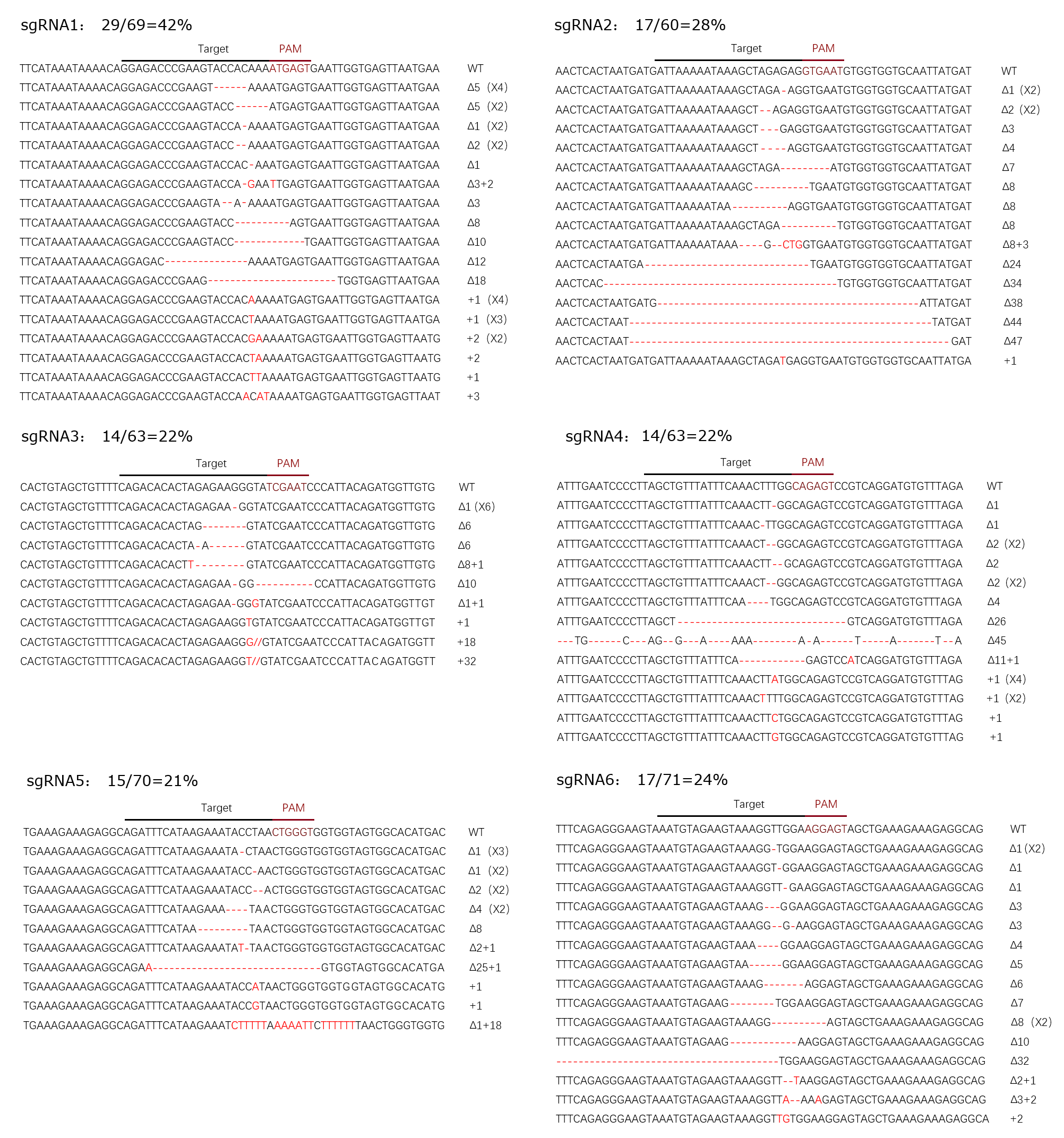


**Figure S2.** Evaluation of the sgRNAs. The cutting activity of the sgRNAs was tested in B16-F10 cells.


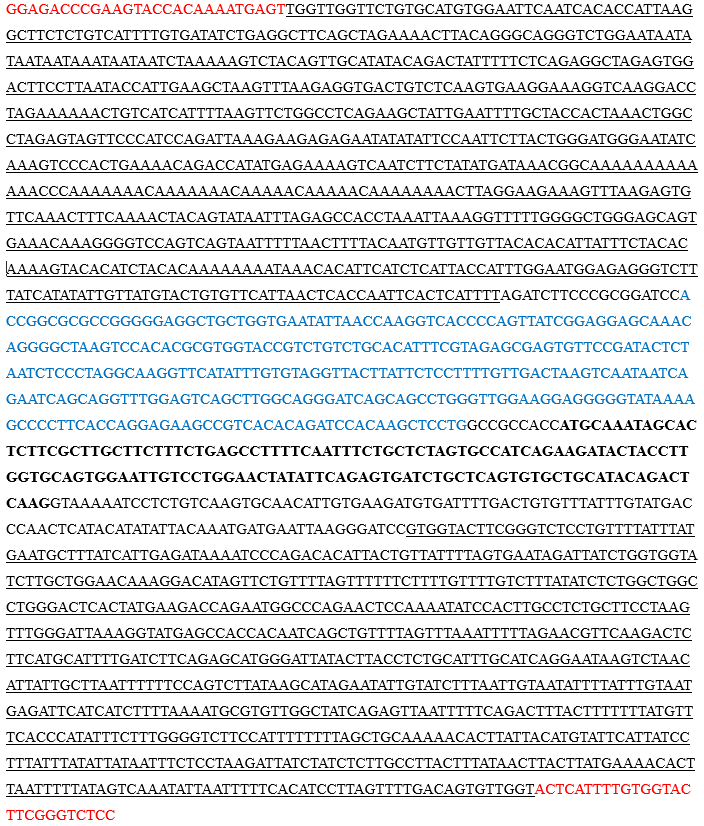
 **Figure S3.** P3 donor, type 2 (5'-3'). Red: sgRNA1 target site; underline: homology sequence; blue: P3 promoter; bold: *F8* exon1. The only difference between type 1 and type 2 donor is that only type 2 donor has the two sgRNA sites.


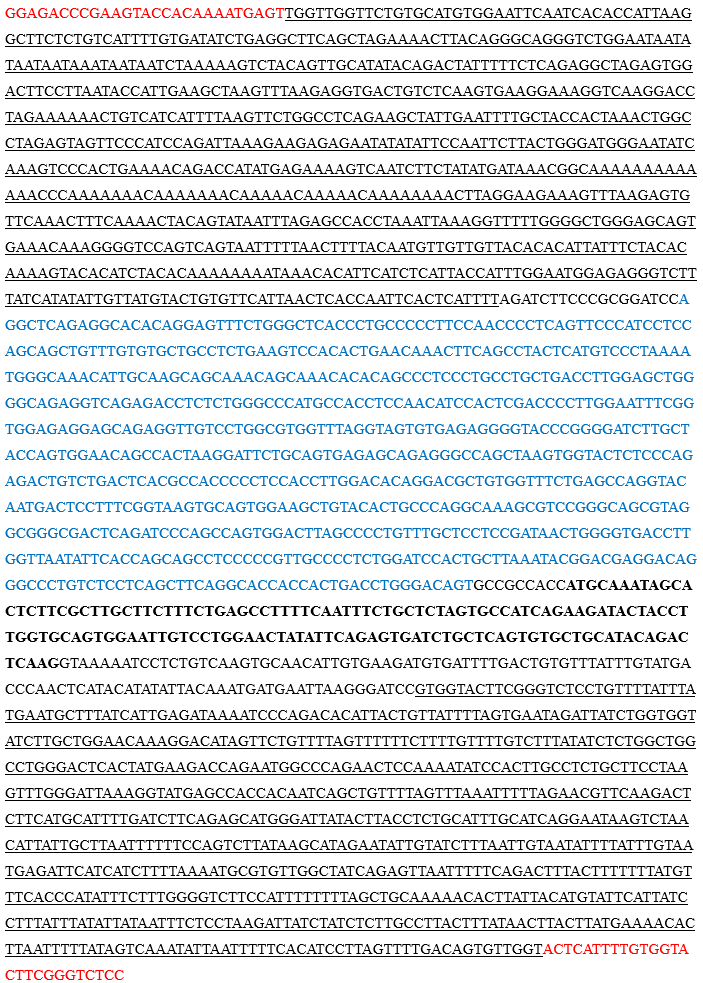
 **Figure S4.** hAAT donor, type 2 (5'-3'). Red: sgRNA1 target site; underline: homology sequence; blue: hAAT promoter; bold: *F8* exon1. The only difference between type 1 and type 2 donor is that only type 2 donor has the two sgRNA sites.


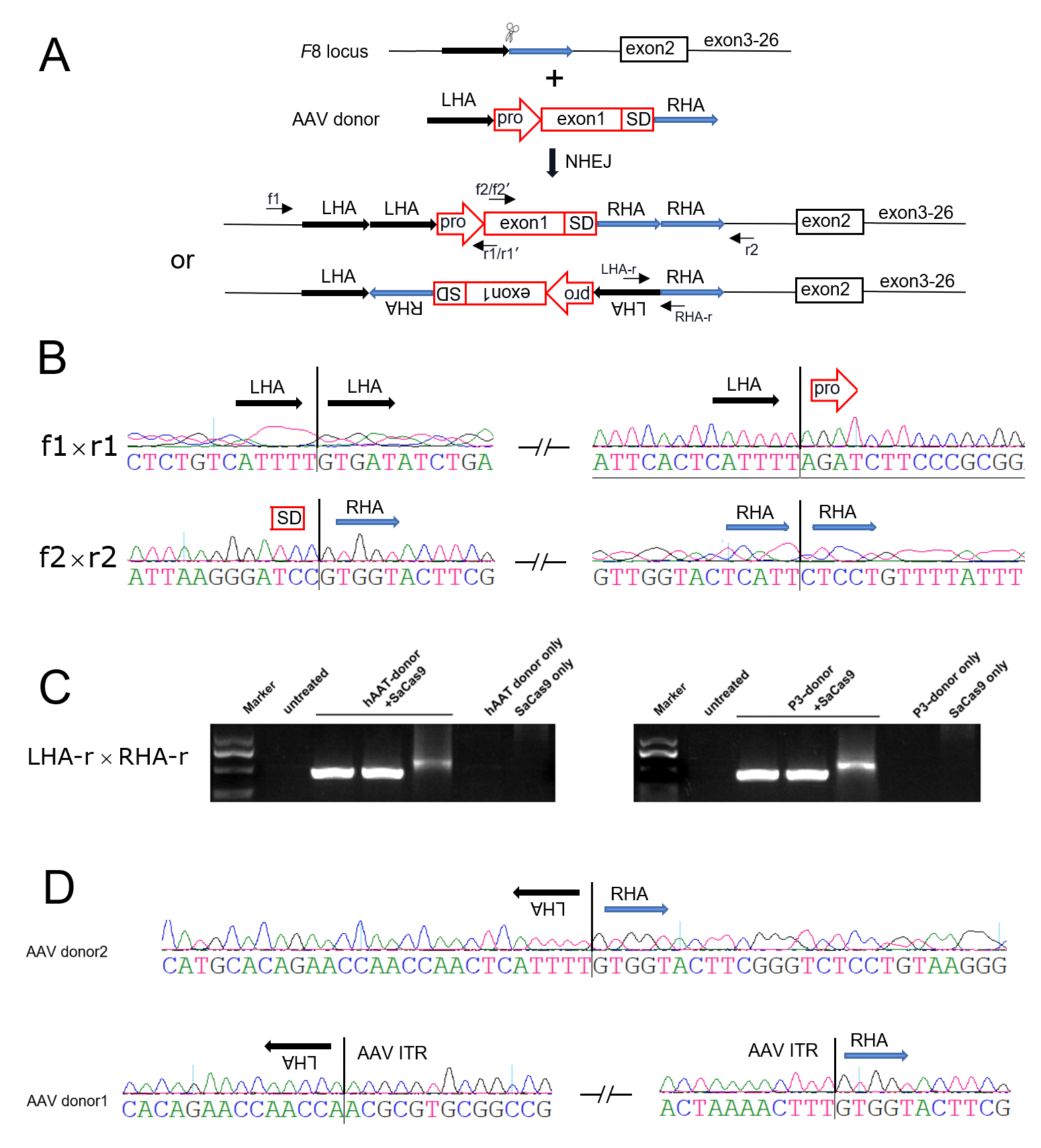


**Figure S5.** Donor integration via NHEJ. **(A)** Schematic of forward and reverse integration of donor vector at the target site via NHEJ. **(B)** Sanger sequencing of the NHEJ band in Figure 4C. **(C)** PCR detection of the reverse integration of donor vectors. For type 1 donors, the AAV ITR sequence will be integrated too, resulting in a larger PCR product. **(D)** Sanger sequencing of the PCR products verified the reverse integration.


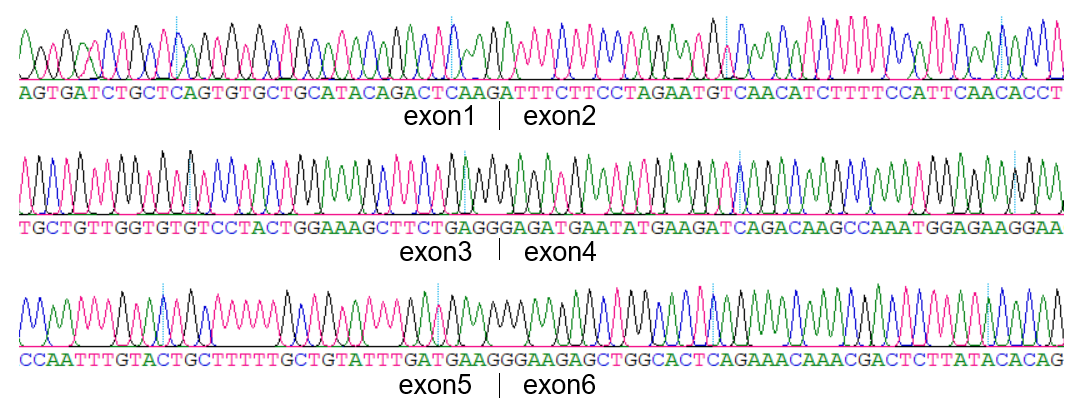
 **Figure S6.** Sanger sequencing of the products of RT-PCR.

**Data S1.** The residual 6506 bp of intron1 in human Inv1 that is suitable for sgRNA design (5'-3').

ATGTGCTTCCTTGGGTGATTTTACAACTTTTTAAGCACATGAATAAAAATTATTGTCTGCTTTTTTCTCCTTTTTTCTCTCTTTTTTTTCTTGCATGCAGAAAGTTTATAGGAGGGGGATCCTCTGGATCAACACCCATGGGGGAGTGAAGGAAGCAGGATTGGGTAAAAGAAGTTGAGGTGATACAGTCTGAATAAAGGTCAGTCCCACAGGGAGCTCAAGCTAGGATGGCTCTGCAGAGATGTTCTCTATTGAGGCAAGTTGTCTGGACCTATATAGTCCCACCTTTACCAGGAATAAGGCATGAGTTTGGATGTGGTGACTGACTTCAGCCAAAATTGGTTCTCAGAGAGGAATTCAGATTAGAGTTGTCAGCTGTCAGCACACCCAGCAGCTTAGGAAATAAGTGCTTTAGTCCTGAAGCGGTGAATCTGGACACCAGCCTCCACTGTGGTCCACCCTTTCTGCTGCTCAGATTCATTTGCTTCATAAGTTCTGGAATGGTTCCTTCAGGATTCTGATGGGCTCCCTTTCCTGGGGAAACAAAAGAGAAAGGTTTGGAACACAGCACAACTCCTGCTGCTTTAGCTTTTCTTGAGTCTGCAACTGGTACTCATCATCTTCCCCTTCTACTACCCATTCTAGATCCCTCCCCCCACCACCCTCTCAATTCATACTTCTGCTGCTCCAAGTGGGTTACCAGATAAGGTATCTGGTCATCATGCCCTTTGTAAGCCATGACTGTACTTGTCCATTTATCATCAACATTGAACAAGGGAATACTAAGAGATACCCAGGCAGATGACCTGGCTGCCAAATATATTCTCCTCTGTCTCCATTGTGTAATGTCAGCCCTTCCTCCTGATGATCAGGATCGGTTACGCTTGCCAGGATAGTGACTCTTTTCCTTACCTCTTGGTCTCTTAATAGTGATCTGAAGTAATATGATGGCAACCATAGTTTCAAGTTTAATGAGACTCTTGGTGTGTCCACTGGCGGACATATTCCCCTTTTAAGAACCAGTACTTCTAAACTCTCAGAGCCCAGAGTTGGGATAAGAGGCATAGTTTCCCTAATTGGGTCACTGGGAGTAATGGTAGTGTGAGGCTGCTCCTACTTACACTCCTTGTTTTCCAAAACCATATATTTTACTTCGGGACACACCCCACTAAACGATGGCCATTGATCTAGAGTATATACTGCATCCTGCAGGATGGTGCCTCGTCCTTAGGTATTATGTCTAAGCTAGTGCCTCAGCTGTGCCTTCAACAAGCTGTCCTACCACTCTATCAAGTGGGTAGCTTCTGGGTAGTGGATTCCATGGTCATGTATCTTCTTGCTTTAAAGTAGGTCTCTTGGTCTGATGCAGTGTTATGTAAGATCAAATATTCTGTGAGGCCTTGAATGGTGGTGTTTACTGAGGCTCACCAAGCAACTGAATGATTTTCTTGAGAAATGGTGACATATCAGAGCCTTAGCTTTGGTCTCTGTTACTGGCAAGTTGAACATTAACAACATCA

GTAGCTAGATCTGTATTGATGAGTCCATGATGTTGGGCCCATGCATAGCCTCTGCCATGGCTCCTCCTTTCATACATCCATTTTGTAAGAATTGGGGCACCAACAGCAGAGACTGGTAAGCATCAAAGTGGCTAAGTCATTCAGTCTACTTGGTTGTTTAGTGCTGTTTCTGTGGCTGATGTTCTTTTCATTTATTCATTCTGTAATGCGTATCCTTTGTAGATTCAATTTCCTTCTCTCTGAACAACTTCTTTGGATATTTATTGAAAGGCAGGTCTACTGTCAATAAATTCCTTAATTTTTATTGTCTGAGAAAGTTTTTATTTCTCCTTCATTTTTGAAGGATACTTTCACAGAGTTCAGAATTTTAGGTTGACTGTTTTTTCTTTCTCTCAACACTTTAAATATGTCAGTGCACTCTCTTCTTGTTTGCATGGTTTCTGAGGCGAAGTTGGATGTAATTTTTATCTTTGCTCCTCTATGGATAAAGTGTTTTTCCCCATCTGGTTTCTTACAACATTTTTTGTCTTTGATTTTCTGGAGTTTGAATATAATATGCC

TAGGTGTAGGGTTTTTTTATTTGTTTGCTCGTTTGCATCTATCTTTTTTTTTTTTTTTTTTTTTTTGAGACAGAATCTCGCCCTGTCACCCAGGCTGGAGTGCAGTGGCGCGAACTCGGCTCACTGCAAGCTCCACCTCTTGGGTTCACGCCATTCTCCTGCCTCAGCCTCCTGAATAGCTGGGACTACAGGCACCCGCCACCACAGCTGGCTAATTTTTTTTGTATTTTTTAGTAAAGATGGGGTTTCACCATGTTAGCCAGGATGGTCTCGATCTCCTGACCTCGTGATCTGCCCGCCTCGGCCTCCCAAAGTGCTGGGATTACAGGCGTGAGCCACCGCGCCCAGCTGCATCCATCTTCTTTGGTATTCTCTGAGCTTCCTGAATCTGTGGTATGGTGTCTGGTGTCTGACATTAATTTGGGGGAAATTCTCAGTCATTATTGCTTTAAAAATTTGCTCCTATTCATTTCTGCATTCTTCTTCTGGCATTTCCATTATGTGTATGTTACAACTTTTGTAGTTGTTGTTCCACAGTTTTTGGACATTCTGGGCCCATGCATAGCCTCTGCCGTTGTTGTTGGTGTTTTTTTTTTTTTCAGTCTTTTTTTCTCTTTGCTTTTCAGTTTTGATTGGAAGTTTCTATTGTCATGTCCTCAAGCTCAGAGATTCTTTCCTCAGCCATGTCTCATCAACCAATGAGCCCATCAAAGACATTCTTCATCTCTGTTACAGTGTTTTGATCTCTAGCATTTCTTTTTTATTCTGTCTTAGAATTTCCATATTTTTCCTTATGATATACATCTGTCATTGCATGTTGTCTACTTTTTACATTAAAGCCATTAGCATATTAATCATAGTTTAAAAAATTCCTGGTCTGATAATTATGACATTCCTGCCATATTTGACTCTGATTCTGATGCTTGCTTGTTCAGTCTCTTCAAATTTTGTGGGTTTTGCCCTTTAGTGTGCCTTGTAATTTCTTGTTAAAAGGTGGACATGATGTACTGGCTATGAGGAACTACAGTAAATAGGCCCATAGTGATGTGGTGAGATGGAAAGAAAGAAAAGATGCTCAACATCACTAATCATTAGAGAAATATAAATCAAAACTACAAAGAGATACTGCCTCACACCCATTAGGATGGCTACTATCAAAACTAAAACAGAAAATAATGAATGTTGGTGAGGATGTGGAGAAATTGGAACCCTTGTGCACTATTGCAAATGTACAATAGGGCAGTTTCTCTAAAAATTAAACAGAATTACCATATGATCCAGCAACTCGACTTCTGGCTATATATGCAAAAGAACTGAAATGGGGTCTTAAAGAGATATTTATACATCAATTGTATTAGGGTTCTCCAATGAAACAGATCCGTGTGTGTGTGTGTGTGTGTGTGTGTGTGTGTGTAAATATATGCCAATGGTAAAAGTACAGGGTCTGAGTCAGAAGGCAGAACCAAGAATGCTTATGTCCAAGGGCAGGAGAAGACAGATGTCCCAGTTCAAACAGGGAGAGTGAATTTGCCCTTCCTCTGTCTTTTTGTTCTATTCAGGCCCTCAGTGGAATGGATAATGCCCACCCACATTGGTGAGAGTGATCTTCTTTACCAATTCACATGCTATTCTCTTCTGGAAACATCATCACAGACATACCTAGAAATACTGTTTTGCCATGTATCTGGGCATCTCTTAGCCCAGTCAAGTTGACATATAAAATTAACCATCATATCCATGTTTATAAGCAGCATTATTCACAATAGCTAAAATGTGGAAGTACTGCAAGTGTCTATTGACAGATGAATGGATAATCTAAATGTGGTATATACACACAAAGCATTACGAAAGAAGGAAATTCTGACACATGCTACGACATAGATGAACCTTGAGTATATTACGCTAAGTGAAATAAGCCAGTCATGAAAAGACAAATGCTGTATGATTCCACTTATATTGGGTACTTAGAGTAGTCAAAATGATACAGAAAGTAGAATGGTGGATGCCAGAGACTGGAGGAGGTGAGAGTTATTGTTTAATGTTTATACAGTTTCAGTTTACAACATAAAATTAATTCTGGAGGTAGATGGGAGCATATTACACGTTTTCTTTTTGGAGATGGGATCTCACTCTGTTGCCCAGGCTGGAGTGCAGTTTTGTGATCATAGCTCACTGCAGCCTTAACTGTCTGGGCTCAAGTGATCCTCCCACCCCAGGCACCAAAGCAGCTAAGACCACAGGTGCACACCACCACGCCTGGCTAATTATTATTATTTGTAGAGGCCAGGTACTGCTGTGTTGCCCAGGCTGGTGTTGAACCCCTGGGCTCAAGTGATCATCCTGCCCTGGCCTCCCAAAGTACTGGGATTACAGGTGTGTGCCACCACACCTGGCCAGAATATTTAATGCTACTGAACTGTATATTTAAACATGCTTGGGCTGGGCATGGTGCCTCATGCCTATAATCCCAGCACTTTGGGAAGCCAAGGAGGGTGGATTGCTTGAGCCCAGGAGTGTGACACCAGCCTGGGCAACATGGTGAAACCCTGTCTCTACAAAAACATTAACCGGGCATGGTGGCACACGCCTGTAGTCCCACCTACTCTGGCGGCTGAGGCGGGAGGATCACTCGAACCCGGGAGGTGGATCTCGCCACTGCACTCCAGCCTGAGCAACAGAGTGAGACCCTGTCAAAAAAAAAAAAAAAAAAAAAAGATTTACGATAGTAAATTTTATGTGTATTTTACCACAATTATTAAAATTGAAGAAAATCCCCATGGGTCACTGGTTTACCAATTTAGCCATTTTCACCATTTTTAGATTGCATTTTCCTTCTCTATCTCAGCATTCTGACTAAAGGAAAAGCAAAATTTCTTACAAGAATGAATGATTCTCCAGTGTGGCTGCTCCTGAAAGGAACAATGAGACTTTTGTTATTTCACCACAAGACTGAGAAAGAAATAAACAAGTGATAAATGCCCAGACCCCTTCTTATTCATTCCACATCCTGGTAATCTGAGGCATGGTGTGGCTTTTACTCCAAGCATATGAATACTTACAATCAGAAGCTGCCTCTCCTTTCCCTCACTTTATCATTATTGCCATCATTTATGACAATGGAGGGAGTGTCTCACTGGATGATATTTGGGGTTCTTTCTTCAGTGGTCCGCCATACCAAGCATTTATTTGTGCTGTTCTCCATCTTTCACATCTACCACTTTTCAAAAGTCCCTGAGAGTCAATAAGACATGCGACTCGAACAACTGCTTCACATCTAAGTGTATGTCTTTAAAAATCTCTCCTTTTCCATGTTTATGTCTGATGTGTTTTGTTATGGCAAAGTGGCATGTAGTATACGGTTCATAAGTGAATCGATGTATATCTGTGGAGGGCAAATGCTCAATTTTAGATCTACACATGAAAGCTTTTTTCCAGGGCAGGAGCCTTGCTTTCTCCAACTAAGCCTTAATTACCCAAATGCCCATGTGTTGTGCCTTTGGCTTGTTGAAATGATACCAACTGGTAAAATCTGATGGGAGATAAAAGTTAGATGTCCAGAAGCATAAAAGTCAGGAAGAGAGGCTCTTTATGGCATAGAGAAAGCTGCAGAACTGCTCCATGGTGTGCAAAACTCATCAGCACATGAAACTAGTGAGTGGAGATAGTCACCTTCAACCACTTTGTAATTTCTACCAATTCACTTGGGGCCAGTGTCCCTTATTCACCAAGATTCACATTGGTCCTTTTGATTATTTGGCGTGAGGAGGCCCCCAGACATGCTAAACTGTCATGCAGGGAGAGAGGAAAAGTTGGGAACCAGCCTTTGCTTAAAAGGGCTTTGACAATGGAGGCAAATAATAACATTTTGGGAACATTTATATAAGTAATTATATAATTTCTGATTCAGTAGCTGGTTCATGAATAAATGAGTAATTGGGAGTATCTGAGTGGAGACAAGAATGAACATTAATGTAGGACTGATTCTGAATTACAGCTTCTGGCACTAGTAGGTTTCAATATACATAGTCTGCTGAAAGGTAATTTTCTCTGTGATATTCCTGGTATCCCTAAGGAGGGAAGTATAATTTTAGGTACATACTGACAAAATTTGAACACAAGGTACTTAGATGAAAGTTATAGTCTGTTTTAGAGATGAAATTGAAGGATGCAGTAGCTATTGCTTTGGCAAAGGATTTTTGGCTGATGCAGTGTATGGTTATTGATTTTATGATTCTTATCATATGGCACCTAATGTATCAGTTGAGTTGTGAAAACAGTGTTTCGGTTTAATGGATGTTAGGGATTTATATACAACTTCAAATTTGCCTCCTTGCTAATAGTAGAAAAGAGTAGTATGGAAGCATTACTTCCAGCTGCTTTTTGAAGTGTCCACCAAAATGAACGACTTTTTCCTTTCTTCACGCA
